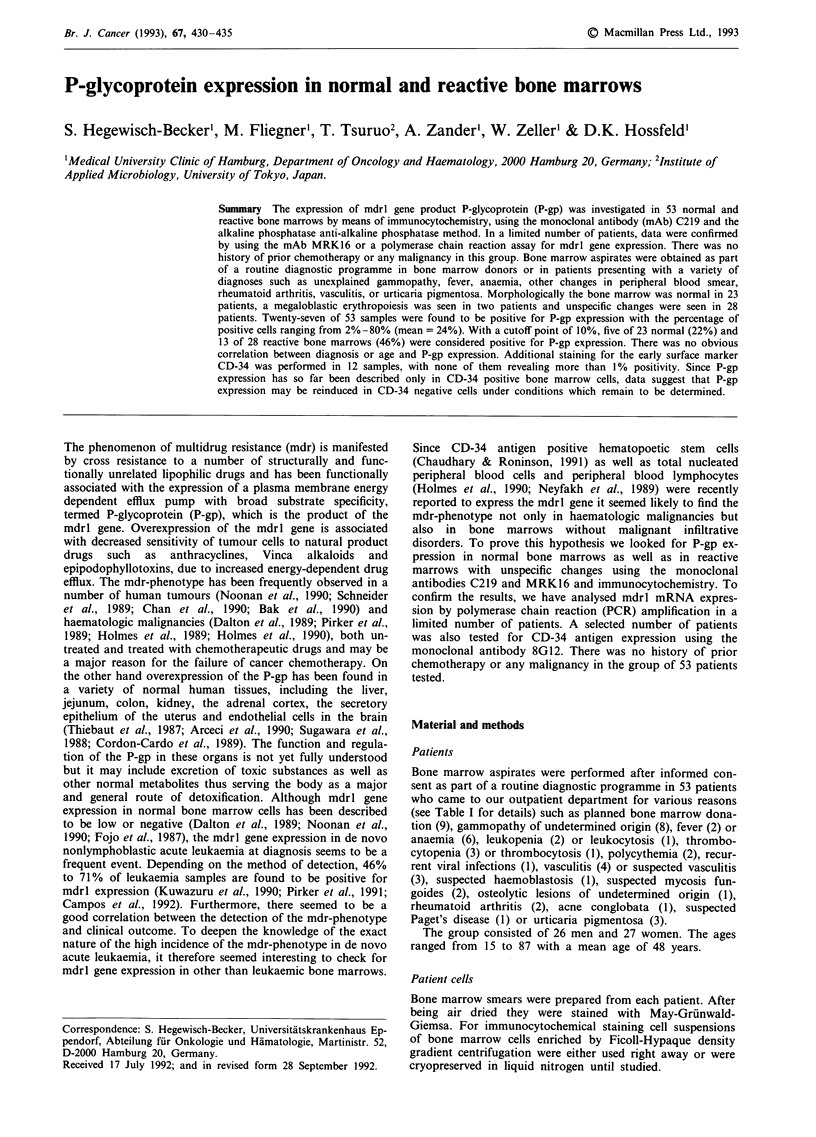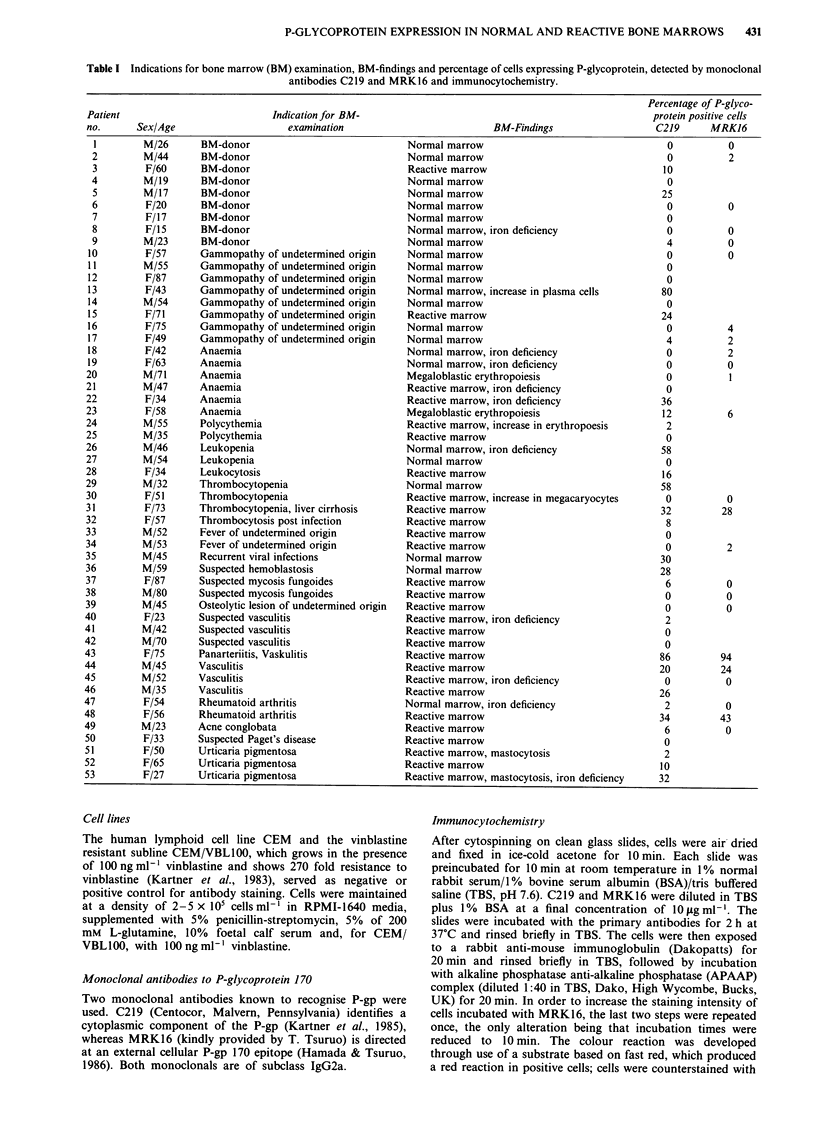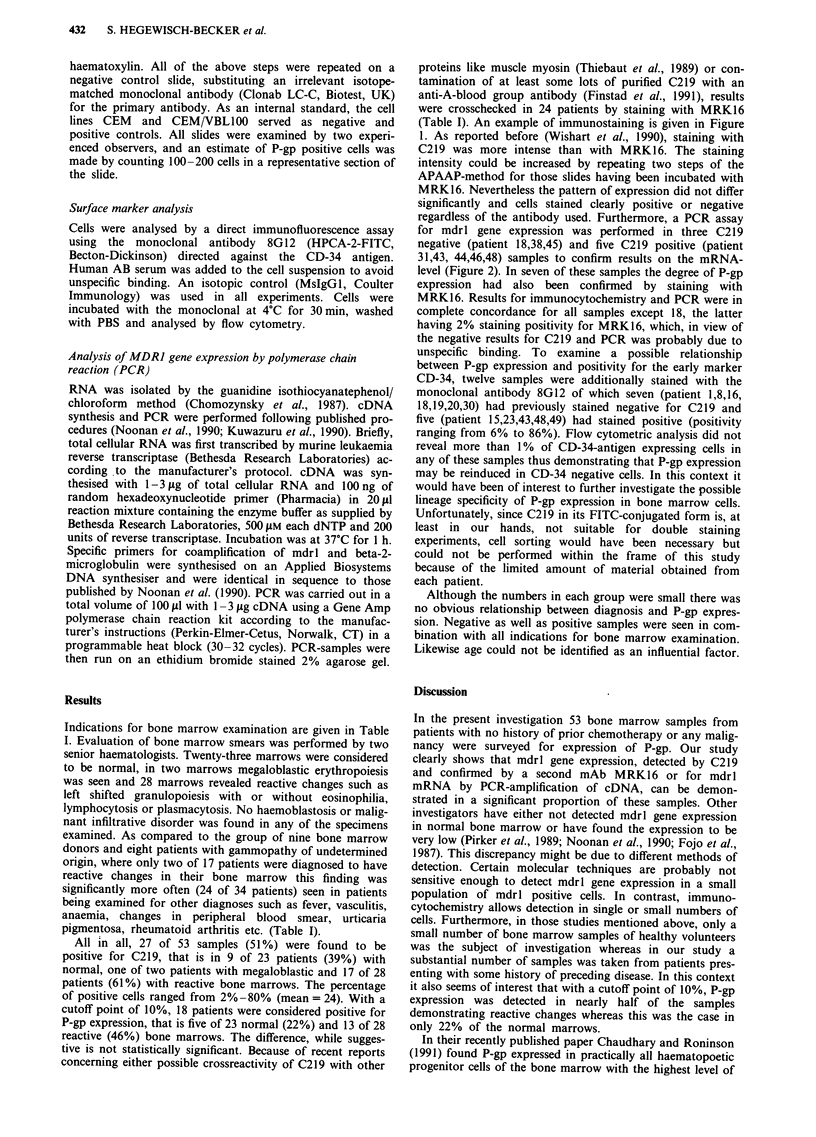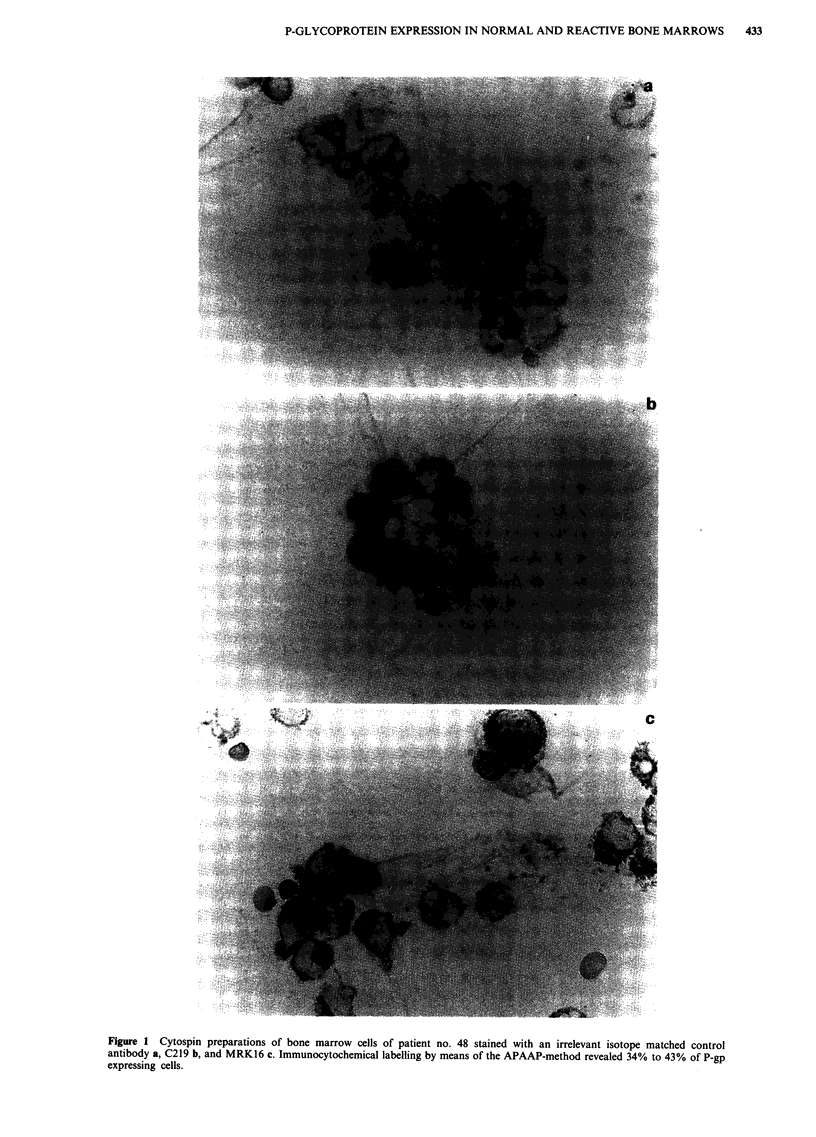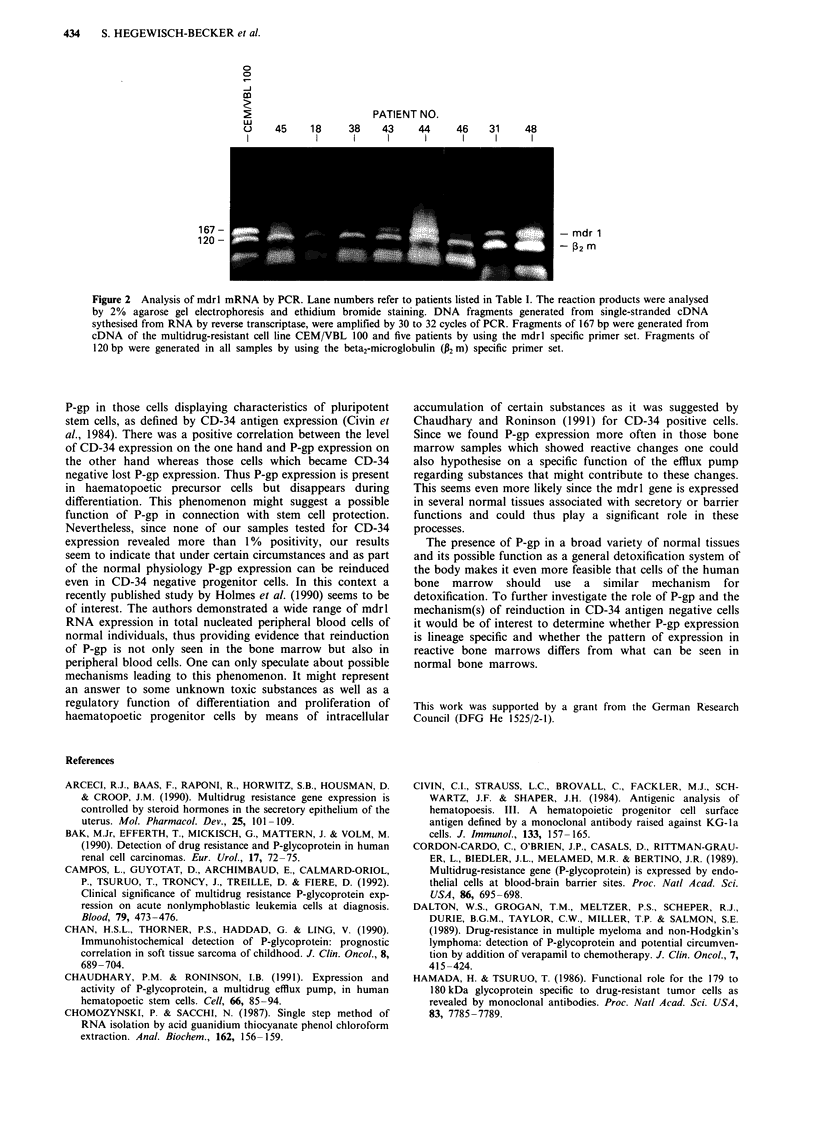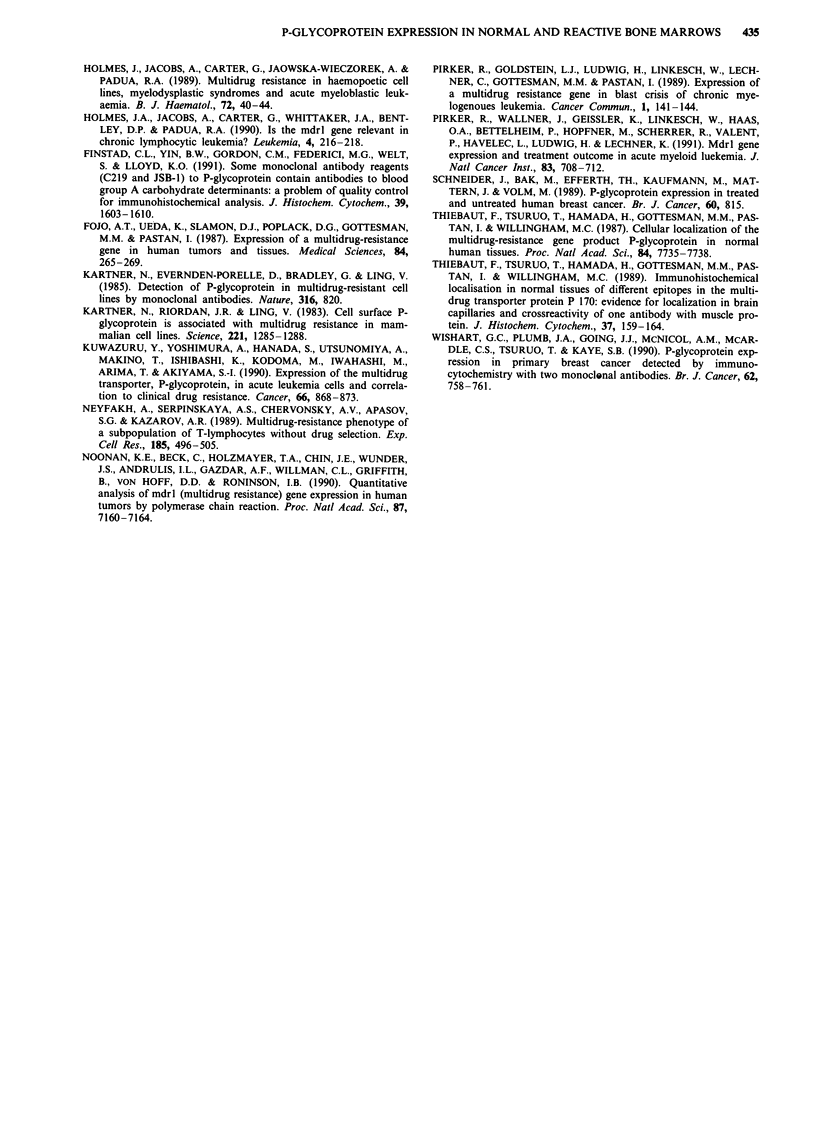# P-glycoprotein expression in normal and reactive bone marrows.

**DOI:** 10.1038/bjc.1993.83

**Published:** 1993-03

**Authors:** S. Hegewisch-Becker, M. Fliegner, T. Tsuruo, A. Zander, W. Zeller, D. K. Hossfeld

**Affiliations:** Medical University Clinic of Hamburg, Department of Oncology and Haematology, Germany.

## Abstract

**Images:**


*Br. J. Cancer* (1993), **67**, 430–435 © Macmillan Press Ltd., 1993

### P-glycoprotein expression in normal and reactive bone marrows

S. Hegewisch-Becker[1], M. Fliegner[1], T. Tsuruo[2], A. Zander[1], W. Zeller[1] & D.K. Hossfeld[1]

[1]*Medical University Clinic of Hamburg, Department of Oncology and Haematology, 2000 Hamburg 20, Germany;* [2]*Institute of Applied Microbiology, University of Tokyo, Japan.*

**Summary** The expression of mdr1 gene product P-glycoprotein (P-gp) was investigated in 53 normal and reactive bone marrows by means of immunocytochemistry, using the monoclonal antibody (mAb) C219 and the alkaline phosphatase anti-alkaline phosphatase method. In a limited number of patients, data were confirmed by using the mAb MRK16 or a polymerase chain reaction assay for mdr1 gene expression. There was no history of prior chemotherapy or any malignancy in this group. Bone marrow aspirates were obtained as part of a routine diagnostic programme in bone marrow donors or in patients presenting with a variety of diagnoses such as unexplained gammopathy, fever, anaemia, other changes in peripheral blood smear, rheumatoid arthritis, vasculitis, or urticaria pigmentosa. Morphologically the bone marrow was normal in 23 patients, a megaloblastic erythropoiesis was seen in two patients and unspecific changes were seen in 28 patients. Twenty-seven of 53 samples were found to be positive for P-gp expression with the percentage of positive cells ranging from 2%–80% (mean = 24%). With a cutoff point of 10%, five of 23 normal (22%) and 13 of 28 reactive bone marrows (46%) were considered positive for P-gp expression. There was no obvious correlation between diagnosis or age and P-gp expression. Additional staining for the early surface marker CD-34 was performed in 12 samples, with none of them revealing more than 1% positivity. Since P-gp expression has so far been described only in CD-34 positive bone marrow cells, data suggest that P-gp expression may be reinduced in CD-34 negative cells under conditions which remain to be determined.

The phenomenon of multidrug resistance (mdr) is manifested by cross resistance to a number of structurally and functionally unrelated lipophilic drugs and has been functionally associated with the expression of a plasma membrane energy dependent efflux pump with broad substrate specificity, termed P-glycoprotein (P-gp), which is the product of the mdr1 gene. Overexpression of the mdr1 gene is associated with decreased sensitivity of tumour cells to natural product drugs such as anthracyclines, Vinca alkaloids and epipodophyllotoxins, due to increased energy-dependent drug efflux. The mdr-phenotype has been frequently observed in a number of human tumours (Noonan *et al.*, 1990; Schneider *et al.*, 1989; Chan *et al.*, 1990; Bak *et al.*, 1990) and haematologic malignancies (Dalton *et al.*, 1989; Pirker *et al.*, 1989; Holmes *et al.*, 1989; Holmes *et al.*, 1990), both untreated and treated with chemotherapeutic drugs and may be a major reason for the failure of cancer chemotherapy. On the other hand overexpression of the P-gp has been found in a variety of normal human tissues, including the liver, jejunum, colon, kidney, the adrenal cortex, the secretory epithelium of the uterus and endothelial cells in the brain (Thiebaut *et al.*, 1987; Arceci *et al.*, 1990; Sugawara *et al.*, 1988; Cordon-Cardo *et al.*, 1989). The function and regulation of the P-gp in these organs is not yet fully understood but it may include excretion of toxic substances as well as other normal metabolites thus serving the body as a major and general route of detoxification. Although mdr1 gene expression in normal bone marrow cells has been described to be low or negative (Dalton *et al.*, 1989; Noonan *et al.*, 1990; Fojo *et al.*, 1987), the mdr1 gene expression in de novo nonlymphoblastic acute leukaemia at diagnosis seems to be a frequent event. Depending on the method of detection, 46% to 71% of leukaemia samples are found to be positive for mdr1 expression (Kuwazuru *et al.*, 1990; Pirker *et al.*, 1991; Campos *et al.*, 1992). Furthermore, there seemed to be a good correlation between the detection of the mdr-phenotype and clinical outcome. To deepen the knowledge of the exact nature of the high incidence of the mdr-phenotype in de novo acute leukaemia, it therefore seemed interesting to check for mdr1 gene expression in other than leukaemic bone marrows.

Correspondence: S. Hegewisch-Becker, Universitätskrankenhaus Eppendorf, Abteilung für Onkologie und Hämatologie, Martinistr. 52, D-2000 Hamburg 20, Germany.
Received 17 July 1992; and in revised form 28 September 1992.

Since CD-34 antigen positive hematopoetic stem cells (Chaudhary & Roninson, 1991) as well as total nucleated peripheral blood cells and peripheral blood lymphocytes (Holmes *et al.*, 1990; Neyfakh *et al.*, 1989) were recently reported to express the mdr1 gene it seemed likely to find the mdr-phenotype not only in haematologic malignancies but also in bone marrows without malignant infiltrative disorders. To prove this hypothesis we looked for P-gp expression in normal bone marrows as well as in reactive marrows with unspecific changes using the monoclonal antibodies C219 and MRK16 and immunocytochemistry. To confirm the results, we have analysed mdr1 mRNA expression by polymerase chain reaction (PCR) amplification in a limited number of patients. A selected number of patients was also tested for CD-34 antigen expression using the monoclonal antibody 8G12. There was no history of prior chemotherapy or any malignancy in the group of 53 patients tested.

### Material and methods

### *Patients*

Bone marrow aspirates were performed after informed consent as part of a routine diagnostic programme in 53 patients who came to our outpatient department for various reasons (see Table I for details) such as planned bone marrow donation (9), gammopathy of undetermined origin (8), fever (2) or anaemia (6), leukopenia (2) or leukocytosis (1), thrombocytopenia (3) or thrombocytosis (1), polycythemia (2), recurrent viral infections (1), vasculitis (4) or suspected vasculitis (3), suspected haemoblastosis (1), suspected mycosis fungoides (2), osteolytic lesions of undetermined origin (1), rheumatoid arthritis (2), acne conglobata (1), suspected Paget's disease (1) or urticaria pigmentosa (3).

The group consisted of 26 men and 27 women. The ages ranged from 15 to 87 with a mean age of 48 years.

### *Patient cells*

Bone marrow smears were prepared from each patient. After being air dried they were stained with May-Grünwald-Giemsa. For immunocytochemical staining cell suspensions of bone marrow cells enriched by Ficoll-Hypaque density gradient centrifugation were either used right away or were cryopreserved in liquid nitrogen until studied.

**Table I** Indications for bone marrow (BM) examination, BM-findings and percentage of cells expressing P-glycoprotein, detected by monoclonal antibodies C219 and MRK16 and immunocytochemistry.

| *Patient no.* | *Sex/Age* | *Indication for BM-examination* | *BM-Findings* | *Percentage of P-glycoprotein positive cells* | |
|---|---|---|---|---|---|
| | | | | *C219* | *MRK16* |
| 1 | M/26 | BM-donor | Normal marrow | 0 | 0 |
| 2 | M/44 | BM-donor | Normal marrow | 0 | 2 |
| 3 | F/60 | BM-donor | Reactive marrow | 10 | |
| 4 | M/19 | BM-donor | Normal marrow | 0 | |
| 5 | M/17 | BM-donor | Normal marrow | 25 | |
| 6 | F/20 | BM-donor | Normal marrow | 0 | 0 |
| 7 | F/17 | BM-donor | Normal marrow | 0 | |
| 8 | F/15 | BM-donor | Normal marrow, iron deficiency | 0 | 0 |
| 9 | M/23 | BM-donor | Normal marrow | 4 | 0 |
| 10 | F/57 | Gammopathy of undetermined origin | Normal marrow | 0 | 0 |
| 11 | M/55 | Gammopathy of undetermined origin | Normal marrow | 0 | |
| 12 | F/87 | Gammopathy of undetermined origin | Normal marrow | 0 | |
| 13 | F/43 | Gammopathy of undetermined origin | Normal marrow, increase in plasma cells | 80 | |
| 14 | M/54 | Gammopathy of undetermined origin | Normal marrow | 0 | |
| 15 | F/71 | Gammopathy of undetermined origin | Reactive marrow | 24 | |
| 16 | F/75 | Gammopathy of undetermined origin | Normal marrow | 0 | 4 |
| 17 | F/49 | Gammopathy of undetermined origin | Normal marrow | 4 | 2 |
| 18 | F/42 | Anaemia | Normal marrow, iron deficiency | 0 | 2 |
| 19 | F/63 | Anaemia | Normal marrow, iron deficiency | 0 | 0 |
| 20 | M/71 | Anaemia | Megaloblastic erythropoiesis | 0 | 1 |
| 21 | M/47 | Anaemia | Reactive marrow, iron deficiency | 0 | |
| 22 | F/34 | Anaemia | Reactive marrow, iron deficiency | 36 | |
| 23 | F/58 | Anaemia | Megaloblastic erythropoiesis | 12 | 6 |
| 24 | M/55 | Polycythemia | Reactive marrow, increase in erythropoesis | 2 | |
| 25 | M/35 | Polycythemia | Reactive marrow | 0 | |
| 26 | M/46 | Leukopenia | Normal marrow, iron deficiency | 58 | |
| 27 | M/54 | Leukopenia | Normal marrow | 0 | |
| 28 | F/34 | Leukocytosis | Reactive marrow | 16 | |
| 29 | M/32 | Thrombocytopenia | Normal marrow | 58 | |
| 30 | F/51 | Thrombocytopenia | Reactive marrow, increase in megacaryocytes | 0 | 0 |
| 31 | F/73 | Thrombocytopenia, liver cirrhosis | Reactive marrow | 32 | 28 |
| 32 | F/57 | Thrombocytosis post infection | Reactive marrow | 8 | |
| 33 | M/52 | Fever of undetermined origin | Reactive marrow | 0 | |
| 34 | M/53 | Fever of undetermined origin | Reactive marrow | 0 | 2 |
| 35 | M/45 | Recurrent viral infections | Normal marrow | 30 | |
| 36 | M/59 | Suspected hemoblastosis | Normal marrow | 28 | |
| 37 | F/87 | Suspected mycosis fungoides | Reactive marrow | 6 | 0 |
| 38 | M/80 | Suspected mycosis fungoides | Reactive marrow | 0 | 0 |
| 39 | M/45 | Osteolytic lesion of undetermined origin | Reactive marrow | 0 | 0 |
| 40 | F/23 | Suspected vasculitis | Reactive marrow, iron deficiency | 2 | |
| 41 | M/42 | Suspected vasculitis | Reactive marrow | 0 | |
| 42 | M/70 | Suspected vasculitis | Reactive marrow | 0 | |
| 43 | F/75 | Panarteriitis, Vaskulitis | Reactive marrow | 86 | 94 |
| 44 | M/45 | Vasculitis | Reactive marrow | 20 | 24 |
| 45 | M/52 | Vasculitis | Reactive marrow, iron deficiency | 0 | 0 |
| 46 | M/35 | Vasculitis | Reactive marrow | 26 | |
| 47 | F/54 | Rheumatoid arthritis | Normal marrow, iron deficiency | 2 | 0 |
| 48 | F/56 | Rheumatoid arthritis | Reactive marrow | 34 | 43 |
| 49 | M/23 | Acne conglobata | Reactive marrow | 6 | 0 |
| 50 | F/33 | Suspected Paget's disease | Reactive marrow | 0 | |
| 51 | F/50 | Urticaria pigmentosa | Reactive marrow, mastocytosis | 2 | |
| 52 | F/65 | Urticaria pigmentosa | Reactive marrow | 10 | |
| 53 | F/27 | Urticaria pigmentosa | Reactive marrow, mastocytosis, iron deficiency | 32 | |

*Cell lines*

The human lymphoid cell line CEM and the vinblastine resistant subline CEM/VBL100, which grows in the presence of 100 ng $ml^{-1}$ vinblastine and shows 270 fold resistance to vinblastine (Kartner *et al.*, 1983), served as negative or positive control for antibody staining. Cells were maintained at a density of $2-5 \times 10^5$ cells $ml^{-1}$ in RPMI-1640 media, supplemented with 5% penicillin-streptomycin, 5% of 200 mM L-glutamine, 10% foetal calf serum and, for CEM/VBL100, with 100 ng $ml^{-1}$ vinblastine.

*Monoclonal antibodies to P-glycoprotein 170*

Two monoclonal antibodies known to recognise P-gp were used. C219 (Centocor, Malvern, Pennsylvania) identifies a cytoplasmic component of the P-gp (Kartner *et al.*, 1985), whereas MRK16 (kindly provided by T. Tsuruo) is directed at an external cellular P-gp 170 epitope (Hamada & Tsuruo, 1986). Both monoclonals are of subclass IgG2a.

*Immunocytochemistry*

After cytospinning on clean glass slides, cells were air dried and fixed in ice-cold acetone for 10 min. Each slide was preincubated for 10 min at room temperature in 1% normal rabbit serum/1% bovine serum albumin (BSA)/tris buffered saline (TBS, pH 7.6). C219 and MRK16 were diluted in TBS plus 1% BSA at a final concentration of 10 $\mu$g $ml^{-1}$. The slides were incubated with the primary antibodies for 2 h at 37°C and rinsed briefly in TBS. The cells were then exposed to a rabbit anti-mouse immunoglobulin (Dakopatts) for 20 min and rinsed briefly in TBS, followed by incubation with alkaline phosphatase anti-alkaline phosphatase (APAAP) complex (diluted 1:40 in TBS, Dako, High Wycombe, Bucks, UK) for 20 min. In order to increase the staining intensity of cells incubated with MRK16, the last two steps were repeated once, the only alteration being that incubation times were reduced to 10 min. The colour reaction was developed through use of a substrate based on fast red, which produced a red reaction in positive cells; cells were counterstained with haematoxylin. All of the above steps were repeated on a negative control slide, substituting an irrelevant isotope-matched monoclonal antibody (Clonab LC-C, Biotest, UK) for the primary antibody. As an internal standard, the cell lines CEM and CEM/VBL100 served as negative and positive controls. All slides were examined by two experienced observers, and an estimate of P-gp positive cells was made by counting 100–200 cells in a representative section of the slide.

*Surface marker analysis*

Cells were analysed by a direct immunofluorescence assay using the monoclonal antibody 8G12 (HPCA-2-FITC, Becton-Dickinson) directed against the CD-34 antigen. Human AB serum was added to the cell suspension to avoid unspecific binding. An isotopic control (MsIgG1, Coulter Immunology) was used in all experiments. Cells were incubated with the monoclonal at 4°C for 30 min, washed with PBS and analysed by flow cytometry.

*Analysis of MDR1 gene expression by polymerase chain reaction (PCR)*

RNA was isolated by the guanidine isothiocyanatephenol/chloroform method (Chomozynsky *et al.*, 1987). cDNA synthesis and PCR were performed following published procedures (Noonan *et al.*, 1990; Kuwazuru *et al.*, 1990). Briefly, total cellular RNA was first transcribed by murine leukaemia reverse transcriptase (Bethesda Research Laboratories) according to the manufacturer's protocol. cDNA was synthesised with 1–3 μg of total cellular RNA and 100 ng of random hexadeoxynucleotide primer (Pharmacia) in 20 μl reaction mixture containing the enzyme buffer as supplied by Bethesda Research Laboratories, 500 μM each dNTP and 200 units of reverse transcriptase. Incubation was at 37°C for 1 h. Specific primers for coamplification of mdr1 and beta-2-microglobulin were synthesised on an Applied Biosystems DNA synthesiser and were identical in sequence to those published by Noonan *et al.* (1990). PCR was carried out in a total volume of 100 μl with 1–3 μg cDNA using a Gene Amp polymerase chain reaction kit according to the manufacturer's instructions (Perkin-Elmer-Cetus, Norwalk, CT) in a programmable heat block (30–32 cycles). PCR-samples were then run on an ethidium bromide stained 2% agarose gel.

### Results

Indications for bone marrow examination are given in Table I. Evaluation of bone marrow smears was performed by two senior haematologists. Twenty-three marrows were considered to be normal, in two marrows megaloblastic erythropoiesis was seen and 28 marrows revealed reactive changes such as left shifted granulopoiesis with or without eosinophilia, lymphocytosis or plasmacytosis. No haemoblastosis or malignant infiltrative disorder was found in any of the specimens examined. As compared to the group of nine bone marrow donors and eight patients with gammopathy of undetermined origin, where only two of 17 patients were diagnosed to have reactive changes in their bone marrow this finding was significantly more often (24 of 34 patients) seen in patients being examined for other diagnoses such as fever, vasculitis, anaemia, changes in peripheral blood smear, urticaria pigmentosa, rheumatoid arthritis etc. (Table I).

All in all, 27 of 53 samples (51%) were found to be positive for C219, that is in 9 of 23 patients (39%) with normal, one of two patients with megaloblastic and 17 of 28 patients (61%) with reactive bone marrows. The percentage of positive cells ranged from 2%–80% (mean = 24). With a cutoff point of 10%, 18 patients were considered positive for P-gp expression, that is five of 23 normal (22%) and 13 of 28 reactive (46%) bone marrows. The difference, while suggestive is not statistically significant. Because of recent reports concerning either possible crossreactivity of C219 with other proteins like muscle myosin (Thiebaut *et al.*, 1989) or contamination of at least some lots of purified C219 with an anti-A-blood group antibody (Finstad *et al.*, 1991), results were crosschecked in 24 patients by staining with MRK16 (Table I). An example of immunostaining is given in Figure 1. As reported before (Wishart *et al.*, 1990), staining with C219 was more intense than with MRK16. The staining intensity could be increased by repeating two steps of the APAAP-method for those slides having been incubated with MRK16. Nevertheless the pattern of expression did not differ significantly and cells stained clearly positive or negative regardless of the antibody used. Furthermore, a PCR assay for mdr1 gene expression was performed in three C219 negative (patient 18,38,45) and five C219 positive (patient 31,43, 44,46,48) samples to confirm results on the mRNA-level (Figure 2). In seven of these samples the degree of P-gp expression had also been confirmed by staining with MRK16. Results for immunocytochemistry and PCR were in complete concordance for all samples except 18, the latter having 2% staining positivity for MRK16, which, in view of the negative results for C219 and PCR was probably due to unspecific binding. To examine a possible relationship between P-gp expression and positivity for the early marker CD-34, twelve samples were additionally stained with the monoclonal antibody 8G12 of which seven (patient 1,8,16, 18,19,20,30) had previously stained negative for C219 and five (patient 15,23,43,48,49) had stained positive (positivity ranging from 6% to 86%). Flow cytometric analysis did not reveal more than 1% of CD-34-antigen expressing cells in any of these samples thus demonstrating that P-gp expression may be reinduced in CD-34 negative cells. In this context it would have been of interest to further investigate the possible lineage specificity of P-gp expression in bone marrow cells. Unfortunately, since C219 in its FITC-conjugated form is, at least in our hands, not suitable for double staining experiments, cell sorting would have been necessary but could not be performed within the frame of this study because of the limited amount of material obtained from each patient.

Although the numbers in each group were small there was no obvious relationship between diagnosis and P-gp expression. Negative as well as positive samples were seen in combination with all indications for bone marrow examination. Likewise age could not be identified as an influential factor.

### Discussion

In the present investigation 53 bone marrow samples from patients with no history of prior chemotherapy or any malignancy were surveyed for expression of P-gp. Our study clearly shows that mdr1 gene expression, detected by C219 and confirmed by a second mAb MRK16 or for mdr1 mRNA by PCR-amplification of cDNA, can be demonstrated in a significant proportion of these samples. Other investigators have either not detected mdr1 gene expression in normal bone marrow or have found the expression to be very low (Pirker *et al.*, 1989; Noonan *et al.*, 1990; Fojo *et al.*, 1987). This discrepancy might be due to different methods of detection. Certain molecular techniques are probably not sensitive enough to detect mdr1 gene expression in a small population of mdr1 positive cells. In contrast, immunocytochemistry allows detection in single or small numbers of cells. Furthermore, in those studies mentioned above, only a small number of bone marrow samples of healthy volunteers was the subject of investigation whereas in our study a substantial number of samples was taken from patients presenting with some history of preceding disease. In this context it also seems of interest that with a cutoff point of 10%, P-gp expression was detected in nearly half of the samples demonstrating reactive changes whereas this was the case in only 22% of the normal marrows.

In their recently published paper Chaudhary and Roninson (1991) found P-gp expressed in practically all haematopoetic progenitor cells of the bone marrow with the highest level of

**Figure 1** Cytospin preparations of bone marrow cells of patient no. 48 stained with an irrelevant isotope matched control antibody **a**, C219 **b**, and MRK16 **c**. Immunocytochemical labelling by means of the APAAP-method revealed 34% to 43% of P-gp expressing cells.

**Figure 2** Analysis of mdrl mRNA by PCR. Lane numbers refer to patients listed in Table I. The reaction products were analysed by 2% agarose gel electrophoresis and ethidium bromide staining. DNA fragments generated from single-stranded cDNA sythesised from RNA by reverse transcriptase, were amplified by 30 to 32 cycles of PCR. Fragments of 167 bp were generated from cDNA of the multidrug-resistant cell line CEM/VBL 100 and five patients by using the mdrl specific primer set. Fragments of 120 bp were generated in all samples by using the beta$_2$-microglobulin ($\beta_2$ m) specific primer set.

P-gp in those cells displaying characteristics of pluripotent stem cells, as defined by CD-34 antigen expression (Civin *et al.*, 1984). There was a positive correlation between the level of CD-34 expression on the one hand and P-gp expression on the other hand whereas those cells which became CD-34 negative lost P-gp expression. Thus P-gp expression is present in haematopoetic precursor cells but disappears during differentiation. This phenomenon might suggest a possible function of P-gp in connection with stem cell protection. Nevertheless, since none of our samples tested for CD-34 expression revealed more than 1% positivity, our results seem to indicate that under certain circumstances and as part of the normal physiology P-gp expression can be reinduced even in CD-34 negative progenitor cells. In this context a recently published study by Holmes *et al.* (1990) seems to be of interest. The authors demonstrated a wide range of mdrl RNA expression in total nucleated peripheral blood cells of normal individuals, thus providing evidence that reinduction of P-gp is not only seen in the bone marrow but also in peripheral blood cells. One can only speculate about possible mechanisms leading to this phenomenon. It might represent an answer to some unknown toxic substances as well as a regulatory function of differentiation and proliferation of haematopoetic progenitor cells by means of intracellular accumulation of certain substances as it was suggested by Chaudhary and Roninson (1991) for CD-34 positive cells. Since we found P-gp expression more often in those bone marrow samples which showed reactive changes one could also hypothesise on a specific function of the efflux pump regarding substances that might contribute to these changes. This seems even more likely since the mdrl gene is expressed in several normal tissues associated with secretory or barrier functions and could thus play a significant role in these processes.

The presence of P-gp in a broad variety of normal tissues and its possible function as a general detoxification system of the body makes it even more feasible that cells of the human bone marrow should use a similar mechanism for detoxification. To further investigate the role of P-gp and the mechanism(s) of reinduction in CD-34 antigen negative cells it would be of interest to determine whether P-gp expression is lineage specific and whether the pattern of expression in reactive bone marrows differs from what can be seen in normal bone marrows.

This work was supported by a grant from the German Research Council (DFG He 1525/2-1).

### References

ARCECI, R.J., BAAS, F., RAPONI, R., HORWITZ, S.B., HOUSMAN, D. & CROOP, J.M. (1990). Multidrug resistance gene expression is controlled by steroid hormones in the secretory epithelium of the uterus. *Mol. Pharmacol. Dev.*, **25,** 101–109.

BAK, M.Jr, EFFERTH, T., MICKISCH, G., MATTERN, J. & VOLM, M. (1990). Detection of drug resistance and P-glycoprotein in human renal cell carcinomas. *Eur. Urol.*, **17,** 72–75.

CAMPOS, L., GUYOTAT, D., ARCHIMBAUD, E., CALMARD-ORIOL, P., TSURUO, T., TRONCY, J., TREILLE, D. & FIERE, D. (1992). Clinical significance of multidrug resistance P-glycoprotein expression on acute nonlymphoblastic leukemia cells at diagnosis. *Blood*, **79,** 473–476.

CHAN, H.S.L., THORNER, P.S., HADDAD, G. & LING, V. (1990). Immunohistochemical detection of P-glycoprotein: prognostic correlation in soft tissue sarcoma of childhood. *J. Clin. Oncol.*, **8,** 689–704.

CHAUDHARY, P.M. & RONINSON, I.B. (1991). Expression and activity of P-glycoprotein, a multidrug efflux pump, in human hematopoetic stem cells. *Cell*, **66,** 85–94.

CHOMOZYNSKI, P. & SACCHI, N. (1987). Single step method of RNA isolation by acid guanidium thiocyanate phenol chloroform extraction. *Anal. Biochem.*, **162,** 156–159.

CIVIN, C.I., STRAUSS, L.C., BROVALL, C., FACKLER, M.J., SCHWARTZ, J.F. & SHAPER, J.H. (1984). Antigenic analysis of hematopoesis. III. A hematopoietic progenitor cell surface antigen defined by a monoclonal antibody raised against KG-1a cells. *J. Immunol.*, **133,** 157–165.

CORDON-CARDO, C., O'BRIEN, J.P., CASALS, D., RITTMAN-GRAUER, L., BIEDLER, J.L., MELAMED, M.R. & BERTINO, J.R. (1989). Multidrug-resistance gene (P-glycoprotein) is expressed by endothelial cells at blood-brain barrier sites. *Proc. Natl Acad. Sci. USA*, **86,** 695–698.

DALTON, W.S., GROGAN, T.M., MELTZER, P.S., SCHEPER, R.J., DURIE, B.G.M., TAYLOR, C.W., MILLER, T.P. & SALMON, S.E. (1989). Drug-resistance in multiple myeloma and non-Hodgkin's lymphoma: detection of P-glycoprotein and potential circumvention by addition of verapamil to chemotherapy. *J. Clin. Oncol.*, **7,** 415–424.

HAMADA, H. & TSURUO, T. (1986). Functional role for the 179 to 180 kDa glycoprotein specific to drug-resistant tumor cells as revealed by monoclonal antibodies. *Proc. Natl Acad. Sci. USA*, **83,** 7785–7789.

HOLMES, J., JACOBS, A., CARTER, G., JAOWSKA-WIECZOREK, A. & PADUA, R.A. (1989). Multidrug resistance in haemopoetic cell lines, myelodysplastic syndromes and acute myeloblastic leukaemia. *B. J. Haematol.*, **72**, 40–44.

HOLMES, J.A., JACOBS, A., CARTER, G., WHITTAKER, J.A., BENTLEY, D.P. & PADUA, R.A. (1990). Is the mdrl gene relevant in chronic lymphocytic leukemia? *Leukemia*, **4**, 216–218.

FINSTAD, C.L., YIN, B.W., GORDON, C.M., FEDERICI, M.G., WELT, S. & LLOYD, K.O. (1991). Some monoclonal antibody reagents (C219 and JSB-1) to P-glycoprotein contain antibodies to blood group A carbohydrate determinants: a problem of quality control for immunohistochemical analysis. *J. Histochem. Cytochem.*, **39**, 1603–1610.

FOJO, A.T., UEDA, K., SLAMON, D.J., POPLACK, D.G., GOTTESMAN, M.M. & PASTAN, I. (1987). Expression of a multidrug-resistance gene in human tumors and tissues. *Medical Sciences*, **84**, 265–269.

KARTNER, N., EVERNDEN-PORELLE, D., BRADLEY, G. & LING, V. (1985). Detection of P-glycoprotein in multidrug-resistant cell lines by monoclonal antibodies. *Nature*, **316**, 820.

KARTNER, N., RIORDAN, J.R. & LING, V. (1983). Cell surface P-glycoprotein is associated with multidrug resistance in mammalian cell lines. *Science*, **221**, 1285–1288.

KUWAZURU, Y., YOSHIMURA, A., HANADA, S., UTSUNOMIYA, A., MAKINO, T., ISHIBASHI, K., KODOMA, M., IWAHASHI, M., ARIMA, T. & AKIYAMA, S.-I. (1990). Expression of the multidrug transporter, P-glycoprotein, in acute leukemia cells and correlation to clinical drug resistance. *Cancer*, **66**, 868–873.

NEYFAKH, A., SERPINSKAYA, A.S., CHERVONSKY, A.V., APASOV, S.G. & KAZAROV, A.R. (1989). Multidrug-resistance phenotype of a subpopulation of T-lymphocytes without drug selection. *Exp. Cell Res.*, **185**, 496–505.

NOONAN, K.E., BECK, C., HOLZMAYER, T.A., CHIN, J.E., WUNDER, J.S., ANDRULIS, I.L., GAZDAR, A.F., WILLMAN, C.L., GRIFFITH, B., VON HOFF, D.D. & RONINSON, I.B. (1990). Quantitative analysis of mdrl (multidrug resistance) gene expression in human tumors by polymerase chain reaction. *Proc. Natl Acad. Sci.*, **87**, 7160–7164.

PIRKER, R., GOLDSTEIN, L.J., LUDWIG, H., LINKESCH, W., LECHNER, C., GOTTESMAN, M.M. & PASTAN, I. (1989). Expression of a multidrug resistance gene in blast crisis of chronic myelogenoues leukemia. *Cancer Commun.*, **1**, 141–144.

PIRKER, R., WALLNER, J., GEISSLER, K., LINKESCH, W., HAAS, O.A., BETTELHEIM, P., HOPFNER, M., SCHERRER, R., VALENT, P., HAVELEC, L., LUDWIG, H. & LECHNER, K. (1991). Mdrl gene expression and treatment outcome in acute myeloid luekemia. *J. Natl Cancer Inst.*, **83**, 708–712.

SCHNEIDER, J., BAK, M., EFFERTH, TH., KAUFMANN, M., MATTERN, J. & VOLM, M. (1989). P-glycoprotein expression in treated and untreated human breast cancer. *Br. J. Cancer*, **60**, 815.

THIEBAUT, F., TSURUO, T., HAMADA, H., GOTTESMAN, M.M., PASTAN, I. & WILLINGHAM, M.C. (1987). Cellular localization of the multidrug-resistance gene product P-glycoprotein in normal human tissues. *Proc. Natl Acad. Sci.*, **84**, 7735–7738.

THIEBAUT, F., TSURUO, T., HAMADA, H., GOTTESMAN, M.M., PASTAN, I. & WILLINGHAM, M.C. (1989). Immunohistochemical localisation in normal tissues of different epitopes in the multidrug transporter protein P 170: evidence for localization in brain capillaries and crossreactivity of one antibody with muscle protein. *J. Histochem. Cytochem.*, **37**, 159–164.

WISHART, G.C., PLUMB, J.A., GOING, J.J., MCNICOL, A.M., MCARDLE, C.S., TSURUO, T. & KAYE, S.B. (1990). P-glycoprotein expression in primary breast cancer detected by immunocytochemistry with two monoclonal antibodies. *Br. J. Cancer*, **62**, 758–761.